# Relationships between parental responsive feeding and infant appetitive traits: The moderating role of infant temperament

**DOI:** 10.3389/fpsyg.2023.1115274

**Published:** 2023-02-06

**Authors:** Yan Liu, Yan Kong, Zhihui Li, Guanghua Zhang, Lin Wang, Guiling Yu

**Affiliations:** ^1^School of Nursing, Qingdao University, Qingdao, Shandong, China; ^2^Department of Nursing, The Affiliated Women and Children’s Hospital of Qingdao University, Qingdao, China; ^3^Office of President, The Affiliated Women and Children’s Hospital of Qingdao University, Qingdao, China

**Keywords:** responsive feeding, appetitive traits, temperament, infants, parents

## Abstract

**Introduction:**

Between the ages of 6 and 12 months is a crucial stage for children to develop appetitive self-regulation. Evidence suggests that a combination of parental responsive feeding and infant temperament (surgency, effortful control, negative affect) shapes infant appetitive traits (food approach, food avoidance). There is a need for research to explore these relationships, in order to provide guidance for the design of an effective intervention to improve appetitive traits. The objective of the current study was to explore the moderating role of infant temperament in the relationship between parental responsive feeding and infant appetitive traits.

**Methods:**

A total of 616 questionnaires, measuring parental responsive feeding, infant appetitive traits, and infant temperament, were collected from parents with infants aged 6–12 months.

**Results:**

Results revealed that responsive feeding was associated with both food approach and food avoidance. Furthermore, only lower levels of surgency significantly moderated the relationship between responsive feeding and food approach, while responsive feeding was associated with food avoidance irrespective of infant temperament.

**Discussion:**

These findings suggest that a strategy embedding responsive feeding interventions should be adopted to reduce infant food avoidance and low-surgent infant food approach, and interventions that are tailored toward food approach for infants with effortful control, negative affect, or higher levels of surgency should be further sought.

## Introduction

Malnutrition and overnutrition among children are still severe public health challenges (World Health Organization, 2020). The State Council Information Office of the People’s Republic of China (2020) reported that children under the age of six were overweight and obese at a rate of 10.4%, which is rapidly increasing, and 5% of them were underweight in 2020. There is growing evidence connecting children’s appetitive traits, which describe characteristics of eating behaviors, to their weight status (Van Jaarsveld et al., 2011; Quah et al., 2015; Vandyousefi et al., 2022). These traits can be classified as food avoidance (e.g., slower eating rate, food fussiness) and food approach (e.g., faster eating rates, emotional overeating, greater responsiveness to food cues; Farrow and Blissett, 2012). Food avoidance tendencies have been shown to predict lower weight status, whereas food approach tendencies have been shown to predict higher weight status (Quah et al., 2015; Vandyousefi et al., 2022). Given the important predictive role of children’s appetitive traits in the development of their weight status, an effective intervention is needed to improve children’s appetitive traits when targeting underweight or overweight. A biopsychosocial model developed by Russell and Russell (2018) proposes that the development of children’s appetitive traits depends on a complex interaction between psychosocial factors (e.g., parental feeding styles and practices) and children’s biological foundations (e.g., temperament). However, further research is warranted to clarify these relationships.

During early childhood, parents are responsible for deciding what, when, and how much to feed their children (Schneider-Worthington et al., 2022). Responsive feeding (RF), as a beneficial feeding practice, is defined as providing developmentally appropriate responses to children’s physiological needs that consider the routine, structure, and emotional context provided by parents (Black and Aboud, 2011; Pérez-Escamilla et al., 2017). RF is theorized to encourage children’s appetitive self-regulation (i.e., eating in response to hunger signals and not eating beyond satiation; Karreman et al., 2006; Black and Aboud, 2011). Several studies have examined the relationships between RF and children’s appetitive traits. Parents of children with good appetitive traits have been shown to employ RF more frequently, such as setting mealtime routines, encouraging children to try new foods, modeling healthy eating, and involving children in food choices (Russell et al., 2015; Mallan et al., 2018). Thus, RF may have potential effects on improving children’s appetitive traits.

Research has revealed that individual differences in children, such as temperament, can shape the development of children’s appetitive traits (Stifter and Moding, 2018). Rothbart and Bates (2006) define children’s temperament using three broad dispositions: surgency (propensity for being highly impulsive and sociable), effortful control (sometimes called regulation/orienting; less emotional reactivity and increased self-regulation), and negative affect (heightened experience of negative emotions). There is evidence that children with higher levels of surgency were more likely to exhibit food approach and children with higher levels of negative affect and lower levels of effortful control were more likely to exhibit food avoidance (Button et al., 2021). Furthermore, the feeding practices that parents use may vary as a function of children’s temperament. For example, mothers who perceived greater infant surgency or negative affect were less likely to use RF and more likely to use food to calm their infants even when they were not physically hungry (Schneider-Worthington et al., 2022), and parents of children with lower levels of negative affect were more likely to use RF (Searle et al., 2020).

Developmental contextualism suggests that the goodness of fit between individual traits (e.g., temperament) and the contexts (e.g., feeding environment) of individuals’ lives determines whether the contexts promote the development of individuals (Zhang and Chen, 2009; Richardson, 2011). For example, the results from a cross-sectional study suggested that children with lower levels of negative affect were less likely to exhibit food fussiness when parents used RF, such as structured meal settings (Searle et al., 2020). It is possible that the fit between RF and children with special temperamental traits (e.g., lower surgency, lower negative affect, or higher effortful control) may be good and thus these children are more likely to exhibit good appetitive traits, which may indicate that RF may simply be more achievable or more successful in improving children’s appetitive traits in children who possess particular temperament traits.

To our knowledge, prior research has examined the moderating effect of children’s temperament on the interplay between RF and eating, and these studies have focused on food choice. For example, the effect of an intervention that encourages the use of RF (e.g., modeling, nonfood rewards alongside repeated exposure) to raise children’s food enjoyment and intake of a disliked vegetable was moderated by children’s sociability (Holley et al., 2016). However, there is no research that examines the effect of children’s temperament on the associations between RF and children’s appetitive traits. This information is crucial because it could guide the design of effective interventions to improve children’s appetitive traits and the advice offered to parents regarding the use of feeding practices, with the potential to modify these to better suit children’s temperament.

Given that the period between 6 and 12 months is crucial for children to develop appetitive self-regulation, which is a key outcome of RF (Pallewaththa et al., 2021) and a determinant of childhood weight status (Vandyousefi et al., 2022), the sample of this study focuses on infants aged 6–12 months. Therefore, the objective of this study was to examine the moderating effect of infant temperament on the relationships between parental RF and infant appetitive traits. It was hypothesized that (1) more frequent use of RF by parents is associated with less food approach and less food avoidance for infants and (2) these associations are moderated by infant temperament and are strongest for infants who have lower levels of surgency or negative affect or higher levels of effortful control.

## Materials and methods

### Study design, settings, and participants

The data of this study were obtained from a multicenter cross-sectional survey conducted in Shandong province, China, between June 2021 and April 2022. Participants were recruited from Child Health Centers in four cities, namely Jinan, Qingdao, Yantai, and Zaozhuang, which were selected based on their geographical locations within Shandong Province. The eligibility criteria were: (1) Chinese (Mandarin)-speaking parents who had an infant aged 6–12 months and (2) parents who were mainly responsible for their infant’s diets. The exclusion criteria of participants (1) had an infant with chronic illness or congenital diseases that affected appetite and (2) had a serious disability that would prohibit them from completing this study’s tasks, such as blindness, etc.

### Procedures

Prior to the survey, ethics approval to conduct this study was obtained from the Institutional Review Board of the hospital (protocol code: QFELL-YJ-2021-95). In addition, one of the authors contacted Child Health Centers managers to obtain their collaboration and content. Then, four researchers with uniform training recruited participants and collected data onsite. After explaining the purpose of this study and obtaining signed informed consent, researchers asked participants to report infant appetitive traits and temperament, their RF, and demographic characteristics *via* questionnaire. Infant height and weight were measured by medical assistants onsite. Participants received a small gift (e.g., shopping bags, hand cream) as a token of compensation for their participation. Overall, 650 parents participated in this study. However, 34 questionnaires were removed as they were completed incorrectly. A total of 616 questionnaires with no missing values were received.

### Measurements

#### Demographic characteristics

This study evaluated basic demographic characteristics including parent sex, education level, relationship with their infants, infant gender, age, and weight status. Infant weight status was assessed using weight-for-length Z score (WLZ), which was classified as <−2 (underweight), −2 to 2 (normal), and > 2 (overweight; World Health Organization, 2013).

#### Parental RF

Parental RF was reported by parents using the 15-item responsive feeding practices assessment tool (RFPAT; Pallewaththa et al., 2021). Each item was rated on a 5-point Likert scale ranging from 0 (rarely) to 4 (always). The scores were computed by averaging all items, with higher scores representing more frequent use of RF. The Chinese version of the RFPAT has been proven reliable (Cronbach’s α = 0.89) and valid (Liu et al., 2022b). In the current sample, Cronbach’s α coefficient was 0.85.

#### Infant appetitive traits

Infant appetitive traits (food approach and food avoidance) were assessed using the Baby Eating Behavior Questionnaire (BEBQ; Llewellyn et al., 2011). Food approach was assessed with the enjoyment of food and food responsiveness subscales, and ratings were averaged across the 8 items to calculate a score for food approach. Food avoidance was assessed with the slowness in eating and satiety responsiveness subscales, and ratings were averaged across the 7 items to calculate a score for food avoidance. The items were rated on a scale of 1 (never) to 5 (always). Higher scores indicate more frequent exhibitions of the corresponding appetitive traits. The Chinese version of the BEBQ has good internal consistency with a Cronbach’s α coefficient of 0.93 (Zhang et al., 2021). The Cronbach’s α coefficients for the food approach and food avoidance subscales in our sample were 0.88 and 0.85.

#### Infant temperament

Infant Behavior Questionnaire-Revised-Short Form (IBQ-R-SF; Putnam et al., 2014), a seven-point Likert scale ranging from 1 (never) to 7 (always), was used to assess parent-reported infant surgency, effortful control, and negative affect. Surgency was measured by 40 items on the smiling and laughter, activity level, high intensity pleasure, perceptual sensitivity, approach, and vocal reactivity subscales. Effortful control was measured by 26 items in the orienting, cuddliness, soothability, and low-intensity pleasure subscales. Negative affect was measured by 25 items on the distress to limitations, fear, sadness, and falling reactivity (reversed scored) subscales. The scores for surgency, effortful control, and negative affect were calculated by averaging items, with higher scores representing higher levels of the corresponding temperament trait. The IBQ-R-SF has been confirmed reliable and of high internal consistency among the Chinese population (Liu et al., 2022a). In the current study, Cronbach’s α coefficients for the surgency, effortful control, and negative affect subscales were 0.85, 0.90, and 0.89.

### Statistical analysis

All data were analyzed using SPSS software version 26. A *p*-value of 0.05 was considered statistically significant. The demographic characteristics and scale scores were summarized *via* descriptive statistics (frequency, mean, standard deviation). Given that the present study used self-report data, Harman’s single-factor test was performed to examine the potential common method biases (Podsakoff et al., 2003). The results showed that there were 11 factors with eigenvalues greater than 1. These factors accounted for 65.31% of the total variance. Only 11.43% of the total variance was explained by the first factor, which failed to reach the critical criterion of 40% (Zhou and Long, 2004), indicating that the present study had no significant common method bias. Kolmogorov–Smirnov tests indicated that the data of continuous variables were non-normally distributed, and Spearman’s correlation analysis was used to test a correlation matrix among all variables. Prior to the analyses of the moderating effect, all variables were standardized.

Moderation models were examined using the SPSS macros program (PROCESS v3.4.1) with 5,000 bootstrap sampling (Hayes, 2017). The potential moderating effects of temperament between RF and appetitive traits were tested by PROCESS model 1 (Hayes, 2017). Category variables were transformed into dummy variables. The conditional indirect effects at different levels of the moderator (at 1 *SD* below and 1 *SD* above the mean) were examined for their statistical significance using the 95% confidence intervals.

## Results

### Participants’ demographics and correlations among variables

In our sample, the mean age of parents was 35.56 (SD = 5.82) ranging from 23 to 62 years, and the mean age of infants was 7.05 (SD = 2.35) ranging from 6 to 12 months. The characteristics of parents and infants are detailed in Table 1. The mean values and bivariate correlations for all variables are presented in Table 2. RF was negatively correlated with food approach (*r* = −0.42, *p* < 0.01) and food avoidance (*r* = −0.28, *p* < 0.01). Both infant gender and infant weight status were correlated with infant food approach and food avoidance (*r* = −0.16, *r* = 0.39; *r* = 0.18, *r* = −0.25; all *p* < 0.01). Therefore, infant gender and infant weight status served as control covariates in the subsequent analysis.

**Table 1:** Characteristics of parents and infants in this study (*n* = 616).

Variables	Categories	*n* (%)
Relationship with infants	Mother	382 (62.0)
	Father	139 (22.6)
	Grandparents	95 (15.4)
Parent sex	Female	405 (65.7)
	Male	211 (34.3)
Parent age	<30 years	273 (44.3)
	30–40 years	210 (34.1)
	>40 years	133 (21.6)
Parent education level	Graduate degree	40 (6.5)
	Bachelor degree	271 (44.0)
	High school degree	274 (44.5)
	Less than a high school degree	31 (5.0)
Infant gender	Boy	286 (46.4)
	Girl	330 (53.6)
Infant age	6–9 months	356 (57.8)
	10–12 months	260 (42.2)
Infant weight status	WLZ < −2	13 (2.1)
	−2 ≤ WLZ ≤ 2	538 (87.3)
	WLZ > 2	65 (10.6)

WLZ, weight-for-length Z score.

**Table 2:** Descriptive statistics and correlations for all the variables (*n* = 616).

Variables	1	2	3	4	5	6	7	8	9	10	11	12	13
1. Relationship													
2. Parent sex	0.01												
3. Parent age	0.05	0.03											
4. Parent education level	0.06	0.01	−0.09										
5. Infant gender	0.03	0.01	0.06	0.02									
6. Infant age	0.05	0.04	−0.07	−0.05	−0.01								
7. Infant weight status	0.23^**^	−0.02	0.05	−0.07	−0.18^**^	0.06							
8. RF	−0.14^*^	0.13^*^	0.05	−0.09	0.01	0.10	−0.17^**^						
9. Food approach	0.11	−0.08	0.09	0.01	−0.16^**^	−0.03	0.39^**^	−0.42^**^					
10. Food avoidance	0.04	−0.06	0.04	−0.04	0.18^**^	0.01	−0.25^**^	−0.28^**^	−0.21^**^				
11. Surgency	0.09	0.03	0.03	0.02	−0.11	0.04	0.28^**^	−0.28^**^	0.35^**^	−0.03			
12. Effortful control	0.02	0.05	−0.05	−0.03	0.08	0.10	−0.09	0.11	−0.04	−0.08	−0.02		
13. Negative affect	0.01	0.06	0.04	0.05	0.09	0.08	−0.03	−0.13^*^	0.02	0.07	−0.04	−0.16^**^	
Mean	-	-	35.56	-	-	7.05	1.07	2.52	3.12	3.09	4.28	3.78	4.67
SD	-	-	5.82	-	-	2.35	0.64	0.73	0.85	0.67	0.86	0.74	0.64
Mix	-	-	23.00	-	-	6.00	−2.31	1.20	1.25	1.00	1.00	1.00	1.50
Max	-	-	62.00	-	-	12.00	3.52	4.00	5.00	5.00	6.65	6.00	6.20

RF, Responsive Feeding. ^*^p < 0.05, ^**^p < 0.01, two-tailed.

### Testing for the moderation model

The moderation analysis, with parental RF as predictor, infant temperament as moderator, and infant appetitive traits as outcome variables, was examined, controlling for infant gender and infant weight status. As shown in Table 3, the moderation model was supported for infant food approach. RF had significant predictive effects on food approach (*β* = −0.31, *p* < 0.001). Furthermore, there was a significant interaction between parental RF and infant surgency in predicting infant food approach (*β* = 0.24, *p* < 0.001). While the interactions between RF and effortful control (*β* = −0.01, *p* > 0.05) and between RF and negative affect (*β* = −0.04, *p* > 0.05) were not significant (see Supplementary Table S1). The moderation model was not supported for infant food avoidance (see Supplementary Table S2). The interactions between RF and surgency, between RF and effortful control, as well as between RF and negative affect were not significant (*β* = −0.03, *β* = 0.04, *β* = 0.09, all *p* > 0.05).

**Table 3:** Results for the moderating effect of surgency on the relationship between RF and food approach.

Outcome variable: food approach					
*R*	*R* ^2^	*F*	*β*	*SE*	*t*
0.59	0.35	34.15^***^			
Infant gender			−0.11	0.05	−2.45^*^
Infant weight status			0.27	0.05	5.13^***^
RF			−0.31	0.05	−6.47^***^
Surgency			0.17	0.05	3.25^***^
RF × surgency			0.24	0.05	5.03^***^

Boy = 0; girl = 1. ^*^p < 0.05, ^**^p < 0.01, ^***^p < 0.001. R^2^, coefficient of determination; *β*, standardized regression coefficient; SE, standard error; RF, Responsive Feeding.

**Figure 1:**
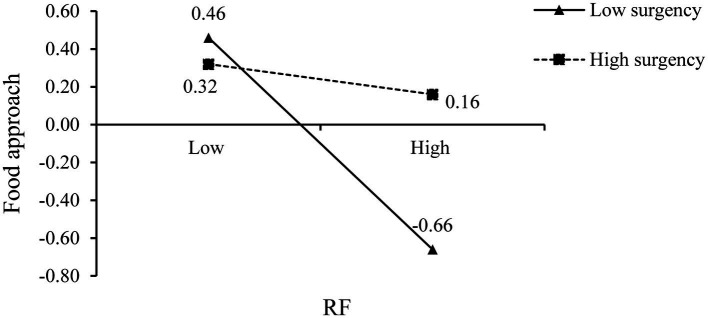
The plot of the relationship between RF and food approach at two levels of surgency. RF, Responsive Feeding. The significant path is represented by the solid line, whereas the non-significant path is represented by the dotted line.

Simple slopes analysis was conducted to further probe the interaction between RF and surgency. The conditional effects were tested at 1 *SD* below (3.42) and 1 *SD* above (5.14) the mean of surgency. The plot of the relationship between RF and food approach at two levels of surgency was described in Figure 1. For infants who were low surgency, parental RF was negatively associated with infant food approach (*β* = −0.55, *SE* = 0.07, 95% *CI* [−0.68, −0.42]), whereas this association was not significant (*β* = −0.14, *SE* = 0.07, 95% *CI* [−0.28, 0.01]) for infants with high surgency.

## Discussion

This study explored the interrelationships between parental RF and infant temperament in shaping infant appetitive traits. In line with our expectations, these findings indicated that RF had significant negative effects on both food approach and food avoidance. Furthermore, only lower levels of surgency significantly moderated the relationship between RF and food approach. In contrast, we did not find support for effortful control or negative affect as significant moderators of this relationship, nor evidence of moderation between RF and food avoidance. Although moderation was limited, this research could be of some value in developing effective interventions that aim to improve infant appetitive traits.

The hypothesized pathways linking parental RF to infant food approach and food avoidance were supported. Parents who reported more frequent use of RF also reported that their infants were less likely to exhibit food approach and food avoidance. The findings are in accordance with previous qualitative research where parents of children with good appetitive traits reported more frequent use of RF (Russell et al., 2015) and confirm a previous quantitative finding in which less food avoidance for infants was associated with more frequent use of RF by mothers (Mallan et al., 2018). Our findings provide evidence that these relationships also exist in infancy, as the sample of previous research focused on toddlers and preschoolers.

As expected, the effect of parental RF on infant appetitive traits varied as a function of infant surgency. Infants whose parents more frequently use RF were less likely to exhibit food approach, and only their low surgency would support this negative impact. The explorative style of surgent infants is outgoing, which reflects a high appetite drive and a propensity to seek pleasure from intaking food in the absence of hunger, and causes infants to be prone to intake food in response to external food signals and to eat at a fast pace (Burton et al., 2011; Leung et al., 2016). However, low-surgent infants are more sensitive to internal cues, such as those of fullness, which may not undermine individual appetitive self-regulation (Burton et al., 2011). RF in response to infant internal cues also fosters the development of infant appetitive self-regulation (Karreman et al., 2006; Black and Aboud, 2011). The combination of RF and low surgency may facilitate the development of infant appetitive self-regulation, which may indicate that for infants with low surgency, parental use of RF is effective in promoting infant appetitive self-regulation and reducing their food approach. Moreover, the findings also coincide with developmental contextualism (Zhang and Chen, 2009; Richardson, 2011). Infants with low surgent temperamental traits may be a good fit for the parental RF context and thus produce positive impacts that infants are less likely to exhibit food approach. Thus, increasing the prevalence of parental RF is conducive to reducing low-surgent infant food approach.

Contrary to our predictions, the relationship between parental RF and infant food approach was not moderated by effortful control. This may be the result of the child’s age since the associations between effortful control and obesogenic appetitive traits were not reported before preschool ages (Leung et al., 2016). Effortful control, the ability to suppress a dominant response to exhibit a subdominant response, consists of a number of self-regulation characteristics, such as inhibitory control, which begin to emerge and mature at the end of the first year of life (Leung et al., 2014). This may indicate that effortful control is not sufficiently developed to regulate obesogenic appetitive traits during infancy, thus, this relationship was not mediated by effortful control. Replicating this study in preschooler samples is needed to examine the significance of the moderating effect of child effortful control on this relationship because the preschool years are a time when effortful control begins to consolidate (Rothbart et al., 2000) and independence and autonomy are increasing (McClelland and Morrison, 2003). Furthermore, negative affect was also not a moderator of this relationship. Negative affect is a predictor of obesogenic appetitive traits (Epel et al., 2001), while it is not associated with food approach in this sample. It may be that we examined appetitive traits concurrently and not prospectively. The relations between negative affect and food approach may emerge over time. Indeed, high-negative affect infants whose parents more frequently use food to soothe are related to more frequent food approach at 4 and 10 years of age (Jansen et al., 2019). In light of this, further longitudinal research to explore the moderating effect of negative affect on this relationship is warranted.

There was no evidence to support that the relationship between parental RF and infant food avoidance varied according to levels of infant temperament. It may be that parental RF is a key reason for a lower prevalence of infant food avoidance irrespective of other infant characteristics. The findings suggest that employing RF may be beneficial for all infants to reduce food avoidance. With this in mind, such practices should be encouraged to parents as beneficial feeding practices for facilitating the development of infant appetitive self-regulation and reducing food avoidance.

## Limitations and implications

Some limitations are worth considering in our study. First, this study was limited by its cross-sectional design, which cannot determine the causal sequence of variables. Future longitudinal research is warranted to evaluate the model identified in this paper. Second, both parental RF and infant appetitive traits and temperament were evaluated by parent reports, which may not always correspond to observable behaviors (Blissett et al., 2019) and may be susceptible to response bias (Bergmeier et al., 2015). Future research should consider measuring these variables through interviews or observation. Last, although the phenomenon of intergenerational coparenting is widespread in China (Li and Liu, 2020), only 15.4% of the present sample were grandparents, which may influence sample representativeness. Thus, our findings need to be further examined within larger grandparent sample sizes.

Despite the above limitations, this research exists potential theoretical and practical implications. On the one hand, the current study provides novel insights into the associations between RF and infant appetitive traits by considering the role of infant temperament while also contributing to clarifying the biopsychosocial model of the development of appetitive traits (Russell and Russell, 2018). On the other hand, our findings add to a growing body of literature that indicates that although the majority of RF previously identified may be beneficial for all infants (Karreman et al., 2006; Black and Aboud, 2011; Guttentag et al., 2014; Pérez-Escamilla et al., 2017), the success of parental use of RF to support infant appetitive self-regulation and improve infant appetitive traits, especially food approach, may depend on individual differences in their infants. Furthermore, the present paper contributes to the development of a systematic evidence base for interventions and policies to improve infant appetitive traits. This study emphasized the important impacts of RF and infant temperament on infant appetitive traits. Given that parental feeding practices are modifiable (Harris et al., 2020), a strategy embedding RF intervention could be adopted to improve infant appetitive traits, and infant temperament should be considered when targeting food approach.

## Conclusion

The findings of the current study suggest that parental RF is associated with infant food avoidance irrespective of infant temperament, and infant surgency functions as a critical moderator of the relationship between parental RF and infant food approach. These findings highlight that RF is an essential component of interventions for reducing infant food avoidance and low-surgent infant food approach, and interventions that seek to tackle infant food approach should take infant temperament into consideration, with a more tailored approach to reducing food approach for infants with effortful control, negative affect, or higher levels of surgency in future research.

## Data availability statement

The original contributions presented in the study are included in the article/Supplementary material, further inquiries can be directed to the corresponding author.

## Ethics statement

The studies involving human participants were reviewed and approved by Institutional Review Board of the Affiliated Women and Children’s Hospital of Qingdao University. The patients/participants provided their written informed consent to participate in this study. Written informed consent was obtained from the individual(s) for the publication of any potentially identifiable images or data included in this article.

## Author contributions

YL and YK developed the study design, collected data, drafted the manuscript, and revised the manuscript critically for important intellectual content. ZL, GZ, and LW collected data, performed the statistical analysis, and drafted the manuscript. GY participated in and supervised the data collection, assisted in data collection and analysis, and made important modification to the manuscript. All authors contributed to the article and approved the submitted version.

## Conflict of interest

The authors declare that the research was conducted in the absence of any commercial or financial relationships that could be construed as a potential conflict of interest.

## Publisher’s note

All claims expressed in this article are solely those of the authors and do not necessarily represent those of their affiliated organizations, or those of the publisher, the editors and the reviewers. Any product that may be evaluated in this article, or claim that may be made by its manufacturer, is not guaranteed or endorsed by the publisher.
