# Supplementary material for: Relationships between parental responsive feeding and infant appetitive traits: The moderating role of infant temperament
Source: Front Psychol. 2023 Feb 6;14:1115274. doi: 10.3389/fpsyg.2023.1115274 (PMC9939436; doi:10.3389/fpsyg.2023.1115274)
Supplement: Supplementary file 1 [file Data_Sheet_1.ZIP › Supplementary table/table s1.docx]

**Supplementary Table 1.** The results for the moderating effect of effortful control or

negative affect on the relationship between RF and food approach.

| **Outcomes** | **Predictors** | ***R*** | ***R*^2^** | ***F*** | ***β*** | ***SE*** | ***t*** |
| --- | --- | --- | --- | --- | --- | --- | --- |
| Food approach |  | 0.53 | 0.28 | 24.93^***^ |  |  |  |
|  | Infant gender |  |  |  | -0.13 | 0.05 | -2.68^*^ |
|  | Infant weight status |  |  |  | 0.28 | 0.05 | 5.61^***^ |
|  | RF |  |  |  | -0.36 | 0.05 | -7.27^***^ |
|  | Effortful control |  |  |  | -0.02 | 0.05 | -0.30 |
|  | RF×effortful control |  |  |  | -0.01 | 0.05 | -0.15 |
| Food approach |  | 0.54 | 0.30 | 26.18^***^ |  |  |  |
|  | Infant gender |  |  |  | -0.13 | 0.05 | -2.62^*^ |
|  | Infant weight status |  |  |  | 0.28 | 0.05 | 5.54^***^ |
|  | RF |  |  |  | -0.37 | 0.05 | -7.50^***^ |
|  | Negative affect |  |  |  | 0.08 | 0.05 | 1.72 |
|  | RF×negative affect |  |  |  | -0.04 | 0.05 | -0.77 |

*Note:* boy=0; girl=1. ^*^*p*<0.05, ^**^*p*<0.01, ^***^*p*<0.001. *R^2^*, coefficient of determination; *β*, standardized regression coefficient; *SE*, standard error; *CI*, bootstrap confidence intervals; RF, Responsive Feeding.
