# Supplementary material for: Relationships between parental responsive feeding and infant appetitive traits: The moderating role of infant temperament
Source: Front Psychol. 2023 Feb 6;14:1115274. doi: 10.3389/fpsyg.2023.1115274 (PMC9939436; doi:10.3389/fpsyg.2023.1115274)
Supplement: Supplementary file 1 [file Data_Sheet_1.ZIP › Supplementary table/table s2.docx]

**Supplementary Table 2.** Results for the moderating effects of surgency, effortful control, or negative affect on the relationship between RF and food avoidance.

| **Outcome** | **predictors** | ***R*** | ***R*^2^** | ***F*** | ***β*** | ***SE*** | ***t*** |
| --- | --- | --- | --- | --- | --- | --- | --- |
| Food avoidance |  | 0.40 | 0.15 | 11.82^***^ |  |  |  |
|  | Infant gender |  |  |  | 0.12 | 0.05 | 2.19^*^ |
|  | Infant weight status |  |  |  | -0.21 | 0.06 | -3.55^***^ |
|  | RF |  |  |  | -0.35 | 0.05 | -6.37^***^ |
|  | Surgency |  |  |  | -0.05 | 0.06 | -0.89 |
|  | RF×surgency |  |  |  | -0.03 | 0.06 | -0.54 |
| Food avoidance |  | 0.40 | 0.16 | 11.98^***^ |  |  |  |
|  | Infant gender |  |  |  | 0.11 | 0.05 | 2.15^*^ |
|  | Infant weight status |  |  |  | -0.23 | 0.05 | -4.19^***^ |
|  | RF |  |  |  | -0.33 | 0.05 | -6.17^***^ |
|  | Effortful control |  |  |  | -0.05 | 0.05 | -0.97 |
|  | RF×effortful control |  |  |  | 0.04 | 0.05 | 0.78 |
| Food avoidance |  | 0.41 | 0.17 | 12.62^***^ |  |  |  |
|  | Infant gender |  |  |  | 0.13 | 0.05 | 2.39^*^ |
|  | Infant weight status |  |  |  | -0.21 | 0.05 | -3.95^***^ |
|  | RF |  |  |  | -0.33 | 0.05 | -6.12^***^ |
|  | Negative affect |  |  |  | 0.05 | 0.05 | 0.86 |
|  | RF×negative affect |  |  |  | 0.09 | 0.05 | 1.75 |

*Note:* boy=0; girl=1. ^*^*p*<0.05, ^**^*p*<0.01, ^***^*p*<0.001. *R^2^*, coefficient of determination; *β*, standardized regression coefficient; *SE*, standard error; *CI*, bootstrap confidence intervals; RF, Responsive Feeding.
